# Lovastatin blocks Kv1.3 channel in human T cells: a new mechanism to explain its immunomodulatory properties

**DOI:** 10.1038/srep17381

**Published:** 2015-11-30

**Authors:** Ning Zhao, Qian Dong, Cheng Qian, Sen Li, Qiong-Feng Wu, Dan Ding, Jing Li, Bin-Bin Wang, Ke-fang Guo, Jiang-jiao Xie, Xiang Cheng, Yu-Hua Liao, Yi-Mei Du

**Affiliations:** 1 Research Center of Ion Channelopathy, Institute of Cardiology, Union Hospital, Tongji Medical College, Huazhong University of Science and Technology, Wuhan 430022, China; 2 Institute of Urology, Union Hospital, Tongji Medical College, Huazhong University of Science and Technology, Wuhan 430022, China; 3 Department of anesthesiology, Zhongshan Hospital, Fudan University, Shanghai 200032, China

## Abstract

Lovastatin is a member of Statins, which are beneficial in a lot of immunologic cardiovascular diseases and T cell-mediated autoimmune diseases. Kv1.3 channel plays important roles in the activation and proliferation of T cells, and have become attractive target for immune-related disorders. The present study was designed to examine the block effect of Lovastatin on Kv1.3 channel in human T cells, and to clarify its new immunomodulatory mechanism. We found that Lovastatin inhibited Kv1.3 currents in a concentration- and voltage-dependent manner, and the IC50 for peak, end of the pulse was 39.81 ± 5.11, 6.92 ± 0.95 μM, respectively. Lovastatin also accelerated the decay rate of current inactivation and negatively shifted the steady-state inactivation curves concentration-dependently, without affecting the activation curve. However, 30 μM Lovastatin had no apparent effect on K_Ca_ current in human T cells. Furthermore, Lovastatin inhibited Ca^2+^ influx, T cell proliferation as well as IL-2 production. The activities of NFAT1 and NF-κB p65/50 were down-regulated by Lovastatin, too. At last, Mevalonate application only partially reversed the inhibition of Lovastatin on IL-2 secretion, and the siRNA against Kv1.3 also partially reduced this inhibitory effect of Lovastatin. In conclusion, Lovastatin can exert immunodulatory properties through the new mechanism of blocking Kv1.3 channel.

Lovastatin belongs to the medication family called Statins. Statins are the oral competitive inhibitors of 3-Hydroxy-3-methylglutaryl coenzyme A (HMG-CoA) reductase, which catalyzes the conversion of HMG-CoA to L-Mevalonate, leading to the blockade of cholesterol biosynthesis. Meanwhile, L-Mevalonate is the precursor for many isoprenoid metabolites. Statins would also inhibit the biosynthesis of isoprenoid intermediates such as geranyl and farnesyl pyrophosphate, and then affect the posttranslational prenylation of several important cell-signaling proteins during immune responses^1^,^2^,^3^. Consequently, along with the lipid-lowering effects, Statins have been demonstrated to exert immunomodulatory properties which decrease the risk of cardiovascular events, such as ischemic heart disease^4^,^5^ and atrial fibrillation^6^. In recent years, more research focus on the immunomodulatory effect of Statins in inflammatory autoimmune diseases^7^. Statins have been reported to be beneficial in a lot of T cell-mediated autoimmune diseases, including experimental autoimmune encephalomyelitis^8^, type 1 diabetes^9^, inflammatory arthritis^10^, autoimmune myocarditis^11^, and autoimmune thyroiditis^12^. Furthermore, many reports suggested that Statins inhibited T cell activation by blocking the Mevalonate pathway and reducing the isoprenoid metabolites^9^,^13^,^14^. Particularly, Lovastatin can inhibit the prenylation and posttranslational activation of Rho GTPase, which facilitates T cell migration, and then suppresses the autoimmune damage in encephalomyelitis and retinal inflammatory disease^8^,^15^. Nevertheless, the immunomodulatory mechanism of Lovastatin in the treatment of T cell-mediated inflammatory diseases which has not been clarified completely requires further study.

Recently, the Kv1.3 channel in T cells has been the novel target for the treatment of many T cell-mediated autoimmune diseases^16^. Kv1.3 channel belongs to the Shaker family, which is preferentially expresses in T cells. Along with K_Ca_ channels, Kv1.3 channel regulates the resting membrane potential and provides sustained driving force for the Ca^2+^ influx through Ca^2+^ -release activated Ca^2+^ (CRAC) channel in T cells. Ca^2+^ influx can activate the Ca^2+^ -dependent signal transcription pathway, and then induces the activation, proliferation and IL-2 secretion of T cells^17^,^18^. Blocking Kv1.3 channel can inhibit the Ca^2+^ influx and Ca^2+^ -mediated signal pathway, and then exert inhibitory effects on T cell activation^19^,^20^,^21^,^22^. In many autoimmune animal models such as multiple sclerosis, rheumatoid arthritis, Type I diabetes, the pathogenic T_EM_ (effector memory T) cells were reported to significantly up-regulate Kv1.3 channel expression after activation, therefore, Kv1.3 blockers can selectively inhibit T_EM_ cells and alleviate the immunologic damage^16^,^23^,^24^,^25^,^26^. Accordingly, Kv1.3 blockers were beneficial for T cell-mediated autoimmune diseases.

The present study was undertaken to determine if the immunomodulatory properties of Statins are mediated by blockade of Kv1.3 channel. In our study, we chose Lovastatin as a represent to validate the above hypothesis. Firstly, we found that Lovastatin concentration- and voltage-dependently blocked Kv1.3 channel in human T cells. However, Lovastatin had no effect on K_Ca_ current, Kv1.3 mRNA and protein expression. Furthermore, Lovastatin inhibited the Ca^2+^ influx, Ca^2+^ -activated transcription factors, T cell proliferation and IL-2 secretion. Finally, we applied Mevalonate and Kv1.3-siRNA to investigate the change of IL-2 production. Our research demonstrated for the first time that Lovastatin can block Kv1.3 channel, decrease Ca^2+^ influx and Ca^2+^ -activated transcriptional factors, and then inhibit the activation, proliferation of human T cells. We may conclude that Lovastatin exerts immunomodulatory effect through Kv1.3 channel.

## Results

### Lovastatin blocked Kv1.3 channel currents in human T cells

**Figure 1:**
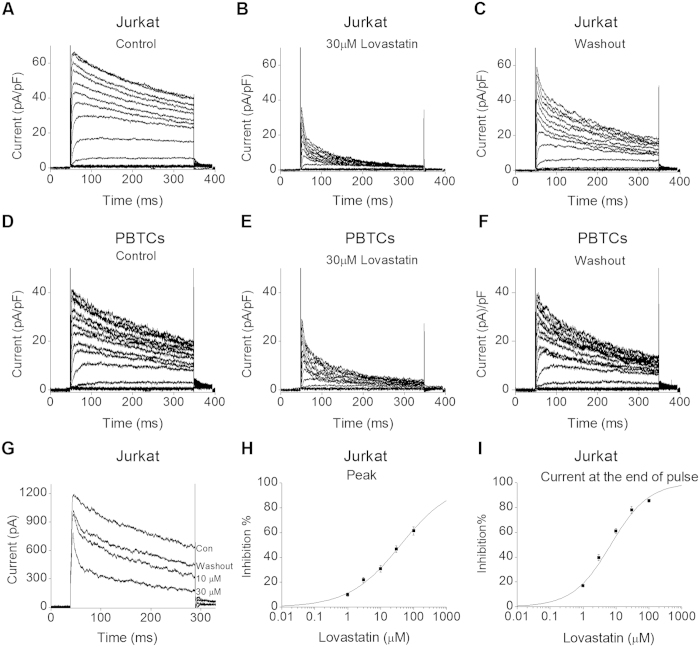
Lovastatin blocked Kv1.3 channel currents in human T cells. (**A–C)** The representative Kv1.3 current traces recorded in Jurkat cells in the absence (**A**), presence (**B**) and washout (**C**) of 30 μM Lovastatin, which were elicited by 300 ms pulses at the test potentials from −80 to + 60 mV in 10 mV steps. (**D–F**) Representative Kv1.3 traces recorded in PBTCs in the absence (**D**), presence (**E**) and washout (**F**) of 30 μM Lovastatin. (**G**) Superimposed original Kv1.3 current traces at control, with 10, 30 μM Lovastatin applications, and washout in Jurkat cells. Kv1.3 currents were elicited by the depolarizing step pulses at + 40 mV with the holding potential at −80 mV every 10 s. (**H**) The dose-response curve of the peak Kv1.3 currents fitted with the Hill equation in Jurkat cells. The summarized data (6 cells) for each concentration are expressed as mean ± SEM. The IC_50_ and Hill coefficient were 39.81 ± 5.11 μM and 0.56 ± 0.04. (**I**) The dose-response curve of Kv1.3 currents at the end of the pulse from 6 Jurkat cells were fitted with the Hill equation. The IC_50_ and Hill coefficient were 6.92 ± 0.95 μM and 0.75 ± 0.06, respectively.

Jurkat cells were held at −80 mV, and received the test potentials from −80 to + 80 mV in 10 mV steps. As shown in Fig. 1A, at test potentials positive to –40 mV, Kv1.3 channel currents rapidly activated and slowly inactivated. However, with 30 μM Lovastatin application, Kv1.3 current was much smaller and inactivated faster (Fig. 1B). After washout, the peak current recovered nearly completely, while the current at the end of the pulse only reversed partially (Fig. 1C). We got the similar results from peripheral blood CD4^+^ T cells (PBTCs, Fig. 1D–F) and CD3^+^ T cells (data not shown). Next, different concentrations of Lovastatin ranging from 1 to 100 μM were applied to block the Kv1.3 channel in Jurkat cells. The current traces in the absence and presence of 10, 30 μM Lovastatin was shown in Fig. 1G. Whereas, the quantitative analysis of concentration-dependent block effect by Lovastatin on the peak and the current at the end of the pulse was shown in Fig. 1H,I, respectively. Using the Hill equation for non-linear fitting, the IC_50_ of Lovastatin on the peak current was calculated to be 39.81 ± 5.11 μM, with the Hill coefficient 0.56 ± 0.04; and the IC_50_ on the current at the end of the pulse was 6.92 ± 0.95 μM, with the Hill coefficient 0.75 ± 0.06.

We further investigated the effects of Lovastatin on cloned human Kv1.3, Kv1.2 and Kv1.5 channels, which were transfected and expressed in HEK 293 cells. As shown in Fig. S1, 30 μM Lovastatin was able to inhibit 83.34 ± 2.04% of Kv1.3 channel peak currents and 93.95 ± 1.48% of currents at the pulse end. Similar blockage effects were also observed in Kv1.2 (70.71 ±1.43%) and Kv1.5 (46.02 ± 1.43% at the peak, 83.95 ± 3.46% at the end of pulse). These results suggested that Lovastatin was a non-selective Kv1.3 blocker.

### The blocking kinetics of Lovastatin on Kv1.3 channel

**Figure 2:**
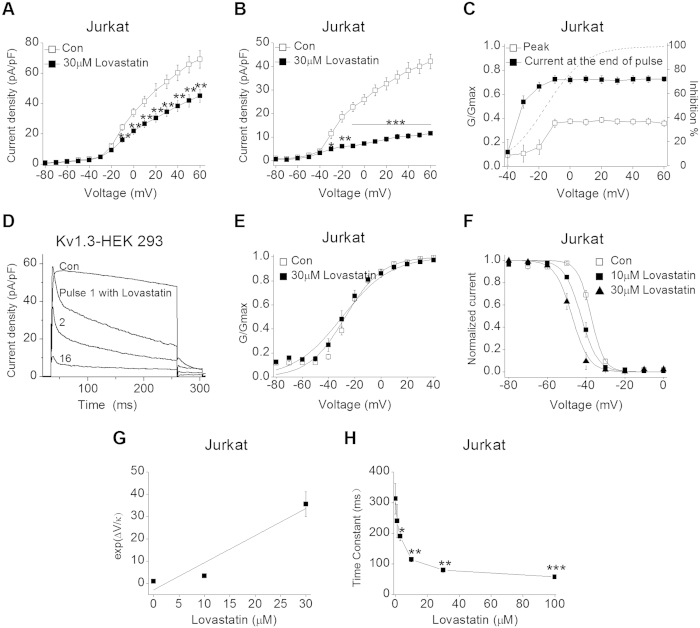
Blocking kinetics of Lovastatin on Kv1.3 channel. (**A**) The summarized current density-voltage relationship for the peak Kv1.3 currents in the absence (open square) and presence (filled square) of 30 μM Lovastatin. Kv1.3 currents were elicited by the same protocol as in Fig. 1A–C. (**B**) The summarized current density-voltage relationship for the end of pulse Kv1.3 currents under control (open square) and in the presence (filled square) of 30 μM Lovastatin. (**C**) The inhibition% of the peak Kv1.3 currents (open square) and currents at the end of pulse (filled square) were plotted with the test voltages. The dashed line showed the fitted activation curve of Kv1.3 currents at control. (**D**) Superimposed current traces recorded before and after a pulse-free period of incubation with Lovastatin. Currents through Kv1.3 channel expressed in HEK 293 were elicited by a 250 ms depolarizing to + 40 mV. Lovastatin (30 μM) was applied to the bath while the membrane potential was held at −80 mV. After an interval of 8 min, consecutive 250 ms pulses were applied every 10 s. The numbers 1 to 16 refers to pulses 1 to 16. (**E**) The activation curves in the absence and presence of 30 μM Lovastatin were fitted with Boltzman equation. G value was defined as *I*/(*V*–E_*rev*_), where *I* was peak amplitude, *V* was corresponding test voltage, and E_*rev*_ was the reversal potential (−90 mV) of Kv1.3 channel. (**F**) The steady-state inactivation curves under control and in the presence of 10, 30 μM Lovastatin. The data were obtained from the normalized currents at + 40 mV, following a 30 s pre-pulse potentials from −80 mV to 0 mV, and fitted with the Boltzman equation to acquire the *V*_*1/2*_ value and *κ*. (**G**) Plot of Exp (∆*V/κ*) value against the Lovastatin concentration. The potential corresponding to half-inactivation voltage *V*_*1/2*_ and slope factor *κ* were acquired from the curves in Fig. 2F. (**H**) The time constants of the Kv1.3 currents decay phase at + 40 mV were acquired with mono-exponential equation and plotted against Lovastatin concentration. (*P < 0.05, **P < 0.01, ***P < 0.001 vs. control).

**Figure 3:**
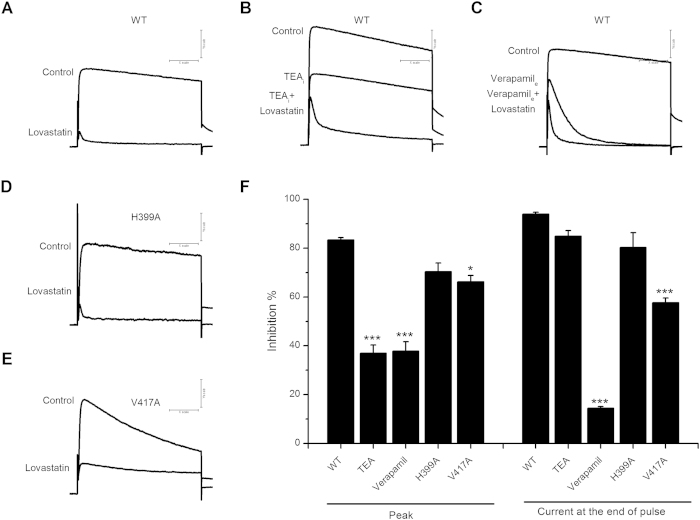
Interaction of Lovastatin with Kv1.3 Wild-Type and Kv1.3 Mutant Channels. Currents were elicited by 200-ms depolarizing voltage steps from a holding potential of −80 to + 40 mV every 10 s. (**A**) Representative currents through WT Kv1.3 channel expressed in HEK 293 cells before and after application of 30 μM Lovastatin. (**B**) Effects of 200 μM internally applied TEA alone and in combination with externally applied 30 μM Lovastatin. (**C**) Effects of of 20 μM externally applied verapamil alone and in combination with externally applied 30 μM Lovastatin. (**D–E**) Representative currents through H399A (**D**) and V417A (**E**) Kv1.3 mutant channels expressed in HEK 293 cells before and after application of 30 μM Lovastatin. (**F**) Average inhibition% of Kv1.3 channel currents by 30 μM Lovastatin. (*P < 0.05, ***P < 0.001 vs. WT).

Next, we observed the voltage-dependent block effect of Lovastatin on Kv1.3 channel. The current density-voltage relationship curves of the peak and the current end of the pulse, before and after 30 μM Lovastatin application, were shown in Fig. 2A,B, respectively. Lovastatin at 30 μM inhibited the peak and the pulse end current apparently above −40 mV when Kv1.3 channel was open. Then, we calculated the inhibition% of 30 μM Lovastatin on the peak and the pulse end current, which was plotted as a function of the test potentials. The activation curve was present in Fig. 3C, too. From –40 mV to 0 mV, the inhibition percentage of Lovastatin on peak and pulse end current increased sharply from 8.7% and 11.0% to 36.8% and 71.1%, respectively. This voltage range was consistent with the voltage for Kv1.3 channel opening. The inhibition increased to a plateau between +10 mV and +60 mV when the channel was fully activated. This blocking character suggested that Lovastatin may bind to the open state of Kv1.3 channel.

We further investigated whether Lovastatin blocked Kv1.3 channel in the closed state. Kv1.3 currents expressed in HEK 293 cells were recorded before and after a pulse-free period of incubation with Lovastatin. Membrane potential was held at −80 mV and Lovastatin (30 μM) was applied to the bath without any depolarization pulses for 8 mins and then the consecutive 250 ms pulses (at + 40 mV) were given every 10 s. As shown in Fig. 2D, Lovastatin (30 μM) did not affect the peak currents at pulse 1 (59.27 vs 55.64 pA/pF), suggesting that little Lovastatin block happened while the channel was closed. However, the onset of blockade occurred rapidly once the channel was opened (the amplitude peak current at pulse 2 was 36.35 vs. 59.27 pA/pF at pulse 1). These results indicated that Lovastatin interacted with the binding sites as the Kv1.3 channel open, with no indication of drug binding in the closed state.

In Fig. 2E, the activation curves in the absence and presence of 30 μM Lovastatin were generated by plotting the normalized conductance with step potentials from −80 mV to + 40 mV using Boltzman equation. Under control, the value of *V*_*1/2*_ for activation was −27.31 ± 1.90 mV and slope factor *κ* was 14.01 ± 1.10; with Lovastatin application, *V*_*1/2*_ was calculated to be −29.57 ± 2.30 mV and *κ* was 18.39 ± 1.33 (P > 0.05). To investigate the effect of Lovastatin on Kv1.3 channel inactivation, in Fig. 2F, we use a standard double-pulse protocol^19^ to generate the steady-state inactivation curves. The resulting normalized current-voltage relationships were fitted using Boltzman equation to acquire the half inactivation voltage *V*_*1/2*_ and *κ*, which were −37.40 ± 0.65 mV and 3.36 ± 0.18 at control. Lovastatin caused a concentration-dependently negative shift of the steady-state inactivation curve. The *V*_*1/2*_ was −42.23 ± 0.99 (P < 0.05 vs. control) with 10 μM Lovastatin, and −47.91 ± 1.55 (P < 0.001) with 30 μM Lovastatin. However, Lovastatin did not alter the *κ* value, which was 4.22 ± 0.25 at 10, 3.04 ± 0.14 at 30 μM Lovastatin (P > 0.05). Then, the *K*_*i*_value of Lovastatin on inactivated-state Kv1.3 channel was calculated to be 0.95 ± 0.21 μM in Fig. 2G. Accordingly, the effect of Lovastatin on channel inactivation was 40 times more potent than on the peak current (39.81 ± 5.11 μM), and 7 times more potent than on the pulse end current (6.92 ± 0.95 μM). Lovastatin may preferentially bind to the inactivated-state of Kv1.3 channel.

Furthermore, the decay phase of Kv1.3 channel current at +40 mV was fitted using a mono-exponential equation, revealing a time constant of 312.52 ± 49.70 ms under control, and 240.26 ± 54.11 (P > 0.05), 190.67 ± 14.95 (P < 0.05), 114.29 ± 7.65 (P < 0.01), 79.79 ± 8.45 (P < 0.01) and 57.73 ± 6.41 (P < 0.001) ms at 1, 3, 10, 30, 100 μM Lovastatin (Fig. 2H). Lovastatin accelerated the channel decay rate in a concentration-dependent manner, similar to many other Kv1.3 open channel blockers^27^,^28^.

### Interaction of Lovastatin with Kv1.3 Wild-Type and Kv1.3 Mutant Channels

To characterize and identify the binding site in Kv1.3 channel that is responsible for the blocking effect of Lovastatin, we firstly performed competition experiments to investigate the interaction of Lovastatin with the known inner pore blockers tetraethyl-ammonium (TEA) and verapamil. Lovastatin (30 μM) caused less blocking effects in the presence of internal TEA (Fig. 3B) or external verapamil (Fig. 3C) than expected for an independent action (Fig. 3A). We then examined the effects of Lovastatin on Kv1.3 H399A and V417A channels, which have been demonstrated to be involved in interactions with small molecular blockers after channel opened. H399A located in the out pore region and V417A located in the S6 domain. As reported, the two mutants can produce substantial outward currents during depolarization^29^. The H399A channel was inhibited by Lovastatin (30 μM) to an extent similar to WT (Fig. 3D), whereas, the inhibitory effect of Lovastatin (30 μM) was significantly less on the V417A channel (Fig. 3E). Figure 3F summarized the inhibitory effect of Lovastatin (30 μM), measured as percentage inhibition of peak and pulse end currents at +40 mV. These results suggested that Lovastatin interacted with the inner pore domain of Kv1.3 channel, and Val417 might be important for the block by Lovastatin.

### Lovastatin did not block K_Ca_ currents in human T cells

**Figure 4:**
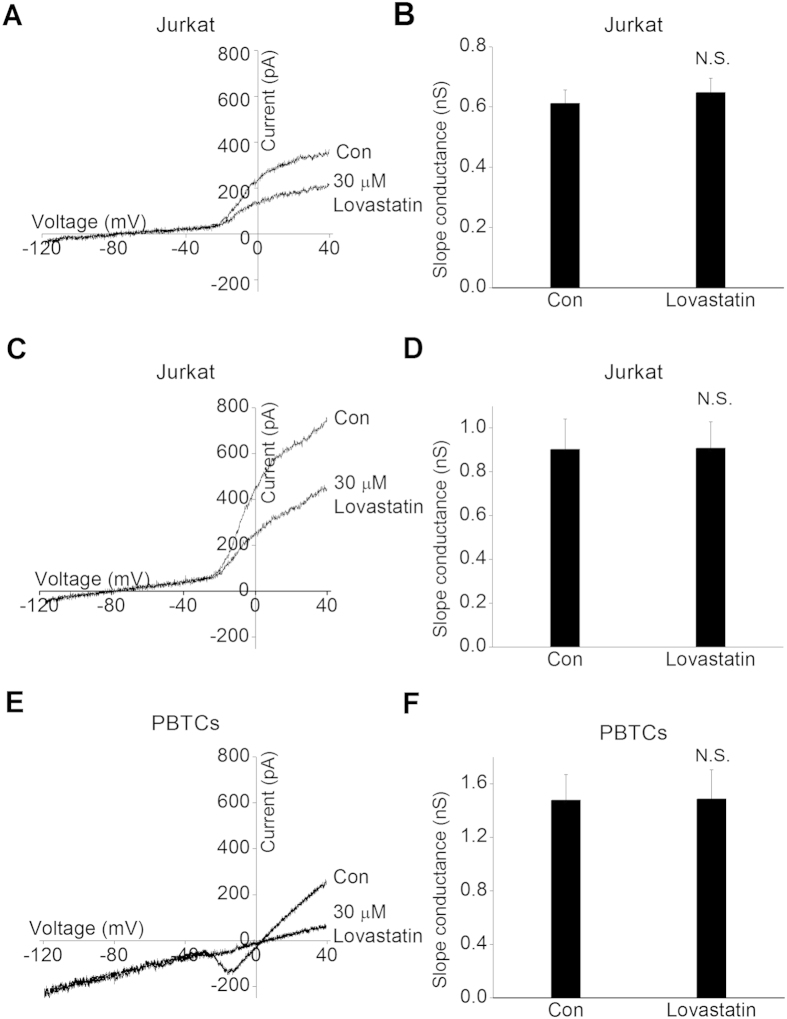
Block effect of Lovastatin on K_Ca_ currents in human T cells. K_Ca_ currents were elicited by 200 ms voltage-ramp from −120 to + 40 mV at the holding potential of −40 mV. The currents slope conductance was acquired by fitting the curves between −120 and −40 mV with linear equation. (**A**) Representative current-voltage relationship traces recorded in the absence or presence of 30 μM Lovastatin in Jurkat cells. (**B**) Summarized slope conductance data from 5 Jurkat cells. (**C**) Representative current-voltage traces of K_Ca_ channel obtained from PBTCs. (**D**) Summarized slope conductance from 5 PBTCs. (**E**) Representative superimposed current-voltage relationships traces from a Jurkat cell recorded in K^+^ Ringer solution with or without 30 μM Lovastatin. (**F**) Summarized data from 5 Jurkat cells. N.S. represented no statistical significance. Data are expressed as the mean ± SEM.

Next, we observed the block effect of Lovastatin on K_Ca_ channel both in Jurkat T cells and PBTCs, because K_Ca_2.2 is the main K_Ca_ channel expressed in Jurkat T cells^30^, whereas K_Ca_3.1 is the main K_Ca_ channel involved in PBTCs^17^. K_Ca_ current was elicited by a 200 ms ramp pulse ranging from −120 mV to + 40 mV at the holding potential of −40 mV. The representative current traces were shown in Fig. 4A,C,E. To avoid the mixture of outward Kv currents, the slope conductance between −120 and −40 mV was measured by fitting the curve with linear equation. As shown in Fig. 4B,D, 30 μM Lovastatin did not block K_Ca_ currents in both Jurkat cells and PBTCs in normal Ringer’s solution. In Jurkat cells, the slope conductance was 0.61 ± 0.05 nS at control, and 0.65 ± 0.05 nS with Lovastatin application (n = 5, P > 0.05). In PBTCs, he slope conductance was 0.90 ± 0.14 nS at control, and 0.91 ± 0.12 nS with Lovastatin application (n = 5, P > 0.05). In Fig. 4E,F, to increase their slope conductance, the K_Ca_ currents were recorded in K^+^ Ringer’s solution, and similarly, 30 μM Lovastatin exerted no block effect on K_Ca_ channel (1.48 ± 0.19 vs. 1.48 ± 0.22 nS, P > 0.05). Consequently, our results suggested that Lovastatin had no apparent block effect on K_Ca_ channel in human T cells.

### Effect of Lovastatin on the expression of Kv1.3 channel in Jurkat cells

**Figure 5:**
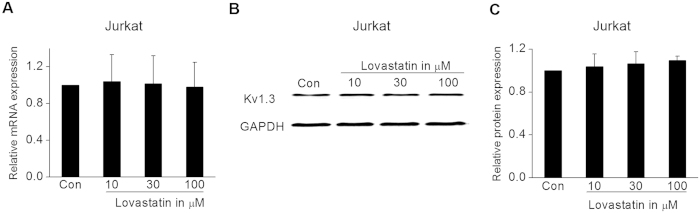
Effect of Lovastatin on Kv1.3 expression in Jurkat cells. (**A**) Jurkat cells were incubated with 10, 30, and 100 μM Lovastatin for 24 h. Then, the relative Kv1.3 mRNA expression level normalized to GAPDH was measured by real-time PCR. The summarized data from 3 replicates was shown. (**B**) Representative Western blotting analysis of Kv1.3 protein expression under control and after 24 h treatment with 10, 30, or 100 μM Lovastatin. (**C**) The summarized data from 3 replicates which were normalized to the protein expression of GAPDH. All the data was expressed as the mean ± SEM.

Besides the acute inhibition of functional Kv1.3 channel, Kv1.3 blockers may exert anti-inflammatory effect through the down-regulation of channel expression^19^,^20^,^31^,^32^. To evaluate the effect of Lovastatin on Kv1.3 expression, Jurkat cells were incubated with 10, 30 and 100 μM Lovastatin for 24 h, and then collected to investigate the Kv1.3 mRNA and protein expression. In Fig. 5A, Lovastatin up to 100 μM performed no apparent effect on Kv1.3 mRNA expression. Similarly, the Kv1.3 protein expression was not changed, either (P > 0.05, Fig. 5).

### The Ca^2+^ influx to Ca^2+^ -depleted Jurkat cells was blunted by Lovastatin

**Figure 6:**
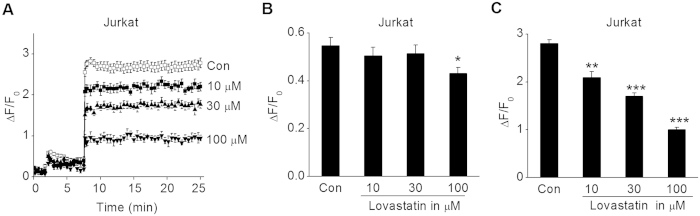
Effect of Lovastatin on the Ca^2+^ influx to Ca^2+^ -depleted Jurkat cells. Jurkat cells were loaded with fluo-4 AM and re-suspended in the Ca^2+^ -free Ringer solution. Then, 10, 30 or 100 μM Lovastatin was applied into the extracellular solution. After 30 min incubation, intracellular Ca^2+^ release and Ca^2+^ influx were elicited by 1 μM TG and 2 mM CaCl_2_, respectively. (**A**) The summarized time course of ΔF/F_0_ value was shown for the cells treated with 0 or 10, 30, 100 μM Lovastatin. (**B**) The summarized ΔF/F_0_ value of the peak intracellular Ca^2+^ release response induced by TG. *P < 0.05 vs. control. (**C**) The summarized ΔF/F_0_ value of the maximum Ca^2+^ influx response after 2 mM CaCl_2_ application. **P < 0.01 and ***P < 0.001 vs. control. All the data are expressed as mean ± SEM from at least 5 replicate experiments.

Many previous studies have shown that Kv1.3 channel plays a vital role in the Ca^2+^ homeostasis of T cells through maintaining the negative membrane potential, and blockade of Kv1.3 channel significantly reduces Ca^2+^ influx^19^,^31^,^33^. As shown in Fig. 6A, in Ca^2+^ -free Ringer’s solution, 1 μM TG caused a small increase of the intracellular Ca^2+^ concentration. After 2 mM CaCl_2_ application, Ca^2+^ flowed into Jurkat cells through CRAC channel and induced significant intracellular Ca^2+^ increase. Incubation with 10 and 30 μM Lovastatin did not alter the Ca^2+^ release induced by TG, but 100 μM Lovastatin slightly inhibited this reaction (P < 0.05, Fig. 6B). In Fig. 6C, 10, 30 and 100 μM Lovastatin concentration-dependently inhibited the Ca^2+^ influx by 25.36% (P < 0.01), 39.29% (P < 0.001) and 64.29% (P < 0.001), respectively.

### Lovastatin down-regulated the Ca^2+^ -related transcription factors NFAT1 and NF-κB p65/50 activities

**Figure 7:**
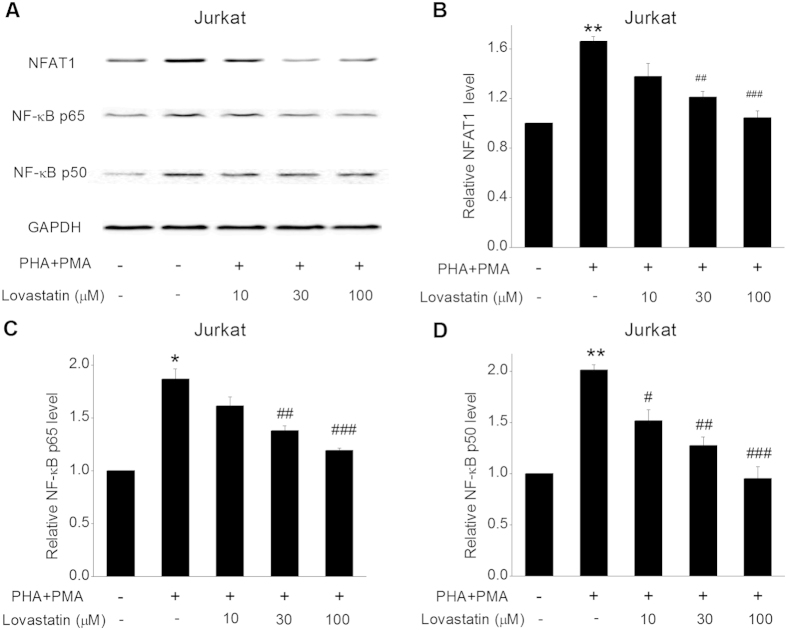
Effect of Lovastatin on the NFAT1 and NF-κB p65/p50 activities. Jurkat cells were pre-treated with 0, 10, 30, or 100 μM Lovastatin for 30 min, then stimulated with PHA + PMA for 4 h. The cells received equal volume of DMSO were defined as control. (**A**) Representative western blotting analysis of NFAT1 and phospho-NF-κB p65/p50 activities. The summarized relative NFAT1 and phospho-NF-κB p65/p50 expression analysis from at least 3 experiments was shown in (**B**) (NFAT1), (**C**) (p65), and (**D**) (p50). *P < 0.05, **P < 0.01 vs. control, ^#^P < 0.05, ^##^P < 0.01 and ^###^P < 0.001 vs. PHA + PMA stimulated group without Lovastatin.

In T cells, Ca^2+^ influx and intracellular Ca^2+^ concentration elevation activate Ca^2+^ -dependent enzyme, and thereby Ca^2+^ -related transcription factors^18^,^34^, so we further investigated the modulation effect of Lovastatin on NFAT1 and NF-κB p65/50. In Fig. 7, the expression of NFAT1 and phospho-NF-κBp65/50 was increased with stimulation by PHA + PMA (P < 0.01 for NFAT1 and p50, P < 0.05 for p65). Treatment with 10, 30 and 100 μM Lovastatin apparently suppressed the expression level of all these three factors concentration-dependently.

### Lovastatin inhibited the proliferation and IL-2 secretion of human T cells

**Figure 8:**
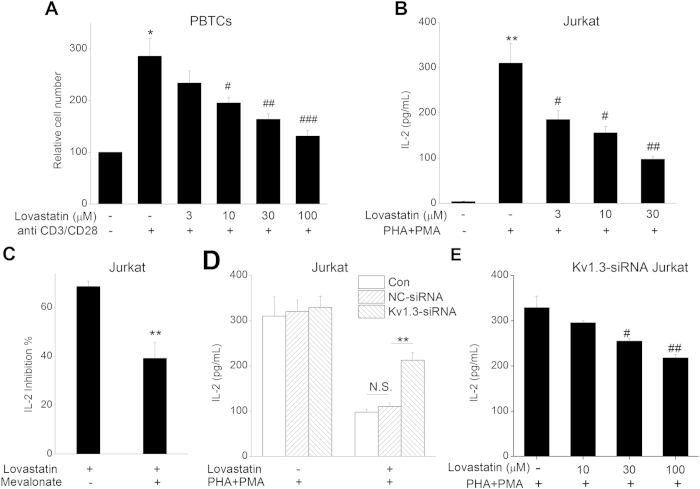
Effect of Lovastatin on T cell proliferation and IL-2 production. (**A**) PBTCs were seeded and pre-incubated with 0, 3, 10, 30, or 100 μM Lovastatin. After 30 min, anti-CD3/CD28 antibodies were added to induce PBTCs proliferation. After 3 days, the relative cell number was determined by CCK-8 kit. The summarized data from at least 5 duplicates was expressed as mean ± SEM. **P < 0.01 vs. control, ^#^P < 0.05, ^##^P < 0.01 vs. CD3/CD28 stimulated group without Lovastatin. (**B**) Jurkat cells were pre-treated with 0, 3, 10, 30, or 100 μM Lovastatin for 30 min, then stimulated with PHA + PMA for 24 h. The supernatants were collected for IL-2 measurement. **P < 0.01 vs. control, ^#^P < 0.05, ^##^P < 0.01 vs. PHA + PMA stimulated group. (**C**) Jurkat cells were treated using 100 μM Lovastatin with or without 1 mM Mevalonate application. Then, the inhibition% of Lovastatin on IL-2 secretion was calculated and showed. (**D**) Con Jurkat cells and NC- or Kv1.3-siRNA-transfected cells were pre-treated with 0 or 100 μM Lovastatin for 30 min, then stimulated with PHA + PMA for 24 h. The supernatants were collected for the measurement of IL-2. **P < 0.01 vs. NC-siRNA group and N.S. represented no statistical significance. (**E**) Kv1.3-siRNA-transfected Jurkat cells were pre-treated with 0, 10, 30, or 100 μM Lovastatin for 30 min, then stimulated with PHA + PMA for 24h. The supernatants were collected for the measurement of IL-2. (^#^P < 0.05, ^##^P < 0.01 vs. PHA + PMA stimulated group).

To investigate the functional immunosuppression effect performed by Lovastatin through inhibiting the Kv1.3 channel, we measured T cell proliferation and IL-2 production. CCK-8 kit was used to access the proliferation of anti CD3/CD28-stimulated human PBTCs, and IL-2 secretion from PHA + PMA activated Jurkat cell was measured by ELISA. In Fig. 8A,B, 3, 10, and 30 μM Lovastatin inhibited the T cell proliferation and IL-2 production concentration-dependently. Since Statins can exert immunomodulatory effect by blocking the isoprenoid pathway^1^,^3^, we applied 1 mM Mevalonate (the intermediate of isoprenoid pathway) along with 30 μM Lovastatin. We found that the blocking effect of Lovastatin on IL-2 secretion was partially neutralized, from 68.57% to 39.11%, which suggested that Lovastatin probably play immunomodulatory effect only partly through isoprenoid pathway. Furthermore, we knocked down Kv1.3 expression using specific Kv1.3-siRNA in Jurkat cells. The Kv1.3-siRNA transfection resulted in reduced inhibitory effect of 30 μM Lovastatin on IL-2 production (34.27 ± 4.74% vs. NC-siRNA 65.94 ± 0.80%, P < 0.01). Simultaneously, NC-siRNA transfection did not alter the block effect of Lovastatin on IL-2 secretion (65.94 ± 0.80% vs. Jurkat cell 68.57 ± 2.20%, P > 0.05). As shown in Fig. 8E, Lovastatin also concentration-dependently reduced IL-2 secretion in Kv1.3 knockdown Jurkat cells. In conclusion, Lovastatin exerted immunomodulatory effect partly through Kv1.3 channel.

## Discussion

This is the first study to investigate the block effect of Lovastatin on Kv1.3 channel in human T cells. Our research demonstrated that Lovastatin blocked Kv1.3 channel in a concentration- and voltage-dependent manner, accelerated the decay rate of current curve and negatively shifted the steady-state inactivation curve without significant effect on K_Ca_ channels. Different from DPO-1, Acacetin and 18β-Glycyrrhetinic acid^19^,^20^,^31^, Lovastatin incubation for 24 h did not change the mRNA and protein expression of Kv1.3 channel, which suggested that Lovastatin may exert its effect by direct blockade. Similar with our previous studies on other Kv1.3 blockers^19^,^20^,^31^, Lovastatin inhibited the Ca^2+^ influx, Ca^2+^ -activated transcription factors NFAT1 and NF-κB p65/50, T cell proliferation and IL-2 production. The Mevalonate pathway is the important mechanism of Statins’ immunomodulatory effect, but Mevalonate application only partially reversed the inhibition of Lovastatin on IL-2 secretion. Furthermore, siRNA against Kv1.3 channel reduced this inhibitory effect of Lovastatin on IL-2 production. Accordingly, Lovastatin can exert anti-inflammatory and immunomodulatory effect partly through Kv1.3 channel in human T cells.

Previous study has proved that Lovastatin blocked Kv1.3 channel in murine thymocytes^35^ without clarifying the blocking characteristic. The IC_50_ of Lovastatin for Kv1.3 current end of the pulse was 6.92 ± 0.95 μM, and the peak plasma Lovastatin bioactivity could reach 12.3 μM^36^. Accordingly, the effective concentration of Lovastatin for Kv1.3 blockade has good clinical relevance. We found that Lovastatin blocked Kv1.3 channel in a voltage-dependent manner, and this effect increased sharply between the voltage ranges from −40 to 0 mV (the voltage of channel open). Moreover, Lovastatin did not produce any obvious effect until the channel was firstly opened by a depolarization pulse, which was similar to the effects of classical Kv channel pore blocker TEA^37^. But, unlike TEA, Lovastatin failed to cause the tail current crossover phenomenon, probably because Lovastatin is not a pure open-channel blocker. Furthermore, in contrast to TEA, Lovastatin concentration-dependently accelerated the decay phase of Kv1.3 channel, and negatively shifted the steady-state curves in a concentration-dependent manner, which indicated that Lovastatin exerted block effect by interacting with inactivated channel, because pure Kv1.3 open channel blockers staurosporine and fluoxetine have no effect on the steady-state inactivated curves^38^,^39^. In conclusion, Lovastatin blocked both the open and inactivated states of Kv1.3 channel, similar with Kv1.3 blockers DPO-1^19^, 18β-Glycyrrhetinic acid^20^, FK-506^28^ and verapamil^40^. We calculated the *K*_*i*_ value for Lovastatin binding to the inactivated Kv1.3 channel, and compared this value with the IC_50_ of Lovastatin on peak or pulse end Kv1.3 current. The *K*_*i*_ value reduced by 40-and 7-fold relatively, which suggested that Lovastatin is much more potent on channel inactivation. This characteristic is very similar to FK-506^28^.

There are not many reports studying the binding sites of Statins and ion channels. Our results of the competitive experiments with well-defined inner-pore blockers TEA and verapamil suggested that Lovastatin interacted with the inner pore of Kv1.3. We further investigated the effects of Lovastatin on the mutant channels (H399A and V417A) and found that V417A significantly reduced the blockade effects of Lovastatin. This particular position has been described previously to be involved in the binding of verapamil to Kv1.3^41^. However, the precise binding sites of Lovastatin require further study.

Kv1.3 is the dominantly expressed K^+^ channel in human T cells, which provides driving force for Ca^2+^ influx by maintaining the resting membrane potential. Blocking Kv1.3 channel can depolarize the membrane potential^42^, decrease Ca^2+^ influx and then inhibit the T cell activation^18^. Along with Kv1.3 channel, T cells also express K_Ca_ channel, which assist to maintain the membrane potential^17^,^43^,^44^. In our study, acute application with Lovastatin significantly inhibited the Ca^2+^ influx into Jurkat cells. But Lovastatin at 30 μM apparently blocked Kv1.3 channel without affecting the K_Ca_ currents. Thus, the effect of Lovastatin on Ca^2+^ influx may attribute to the blocking of Kv1.3 instead of K_Ca_ channels. However, the direct blockade of Lovastatin on CRAC channels (the channel for Ca^2+^ entry) requires further investigation. Ca^2+^ influx can activate the Ca^2+^ -related transcription factors, and ultimately lead to cytokine release and T cell proliferation as well^18^. We also observed that Lovastatin down-regulated the Ca^2+^ -related NFAT1 and NF-κB p65/50 activities. In a previous study, Lovastatin inhibited the IL-2 secretion of human PBTCs in a concentration-dependent manner^45^. Similarly, we found that Lovastatin apparently decreased the IL-2 production of Jurkat cells and PBTCs proliferation. The knockdown of Kv1.3 channel significantly attenuated the block effect of Lovastatin on IL-2 secretion, which indicated that Lovastatin exert anti-inflammatory action by blocking Kv1.3 channnel.

Many previous studies have proved that Lovastatin can down-regulate the serum inflammation mediators IL-6, TNF-α, and CRP^46^,^47^. Lovastatin also performs immunomodulatory function in T cell-mediated autoimmune diseases by inhibiting theisoprenoid and Mevalonate pathway^3^. For example, Lovastatin alleviated the autoimmune retinal disease by inhibiting the isoprenoid pyrophosphate synthesis and activation of GTPase^15^. In experimental autoimmune phalomyelitis model, Lovastatin exerted therapy action through altering the Th1/Th2 cytokines ratio^8^. In a rat model for Guillain–Barre´ syndrome, Lovastatin attenuated the nerve damage by decreasing the proliferation and migration of autoimmune lymphocytes, which can be neutralized by Mevalonate injection^48^. But in our study, we applied Mevalonate in the presence of Lovastatin, and found that the inhibitory action of Lovastatin on IL-2 was only partly neutralized. Therefore, besides the classical Mevalonate pathway, Lovastatin can perform immunodulation function by blocking Kv1.3 channel. In T cell-mediated autoimmune diseases, Lovastatin may possess important therapeutic meanings by blocking Kv1.3 channel and inhibiting the pathogenic action of T_EM_ cells without affecting the physiological immune reactions (which is mediated by the up-regulation of K_Ca_ channels^49^).

In summary, our study proved that HMG-CoA inhibitor Lovastatin can block Kv1.3 channel, decrease Ca^2+^ influx and Ca^2+^ -activated transcriptional factors, and finally inhibit the proliferation, IL-2 secretion of T cells, which is probably a new mechanism for its immunomodulatory effect. Kv1.3 blocker Lovastatin might play important therapeutic roles in T cell-mediated immune diseases.

## Methods

### Ethics statement

Our study protocol of the healthy volunteers’ blood samples was conducted according to the Declaration of Helsinki and Tokyo for humans. The protocol was also approved by the Ethics Committee of Tongji Medical College, Huazhong University of Science and Technology (Approval reference number: IORG0003571). Healthy volunteers were all provided with written informed consent for the collection of blood and the subsequent T cell isolation and analysis.

### Chemicals

Lovastatin was a gift from Prof. Huang (Xiamen, China). Phytohematogglutinin (PHA) and phorbol ester (PMA) were obtained from Sigma-Aldrich (St Louis, MO, USA). Thapsigargin (TG) was obtained from Alomone Laboratories (Jerusalem, Israel), and fluo-4 AM was purchased from Invitrogen (Carlsbad,CA, USA). RPMI 1640, fetal bovine serum, and penicillin/streptomycin were all from Gibco (Grand Island, NY, USA). Lovastatin was dissolved in DMSO at 100 mM stock concentration. All aliquoted stock was kept light protected at −20 °C. During all experiments, the final concentration of DMSO in cell culture medium were no more than 0.1%, and the control with corresponding DMSO was used to discard the possible non-specific effects.

### Cell preparation

The human Jurkat T cell-line was obtained from the China Center for Type Culture Collection (Wuhan, China). Mononuclear cells were separated using the density Ficoll gradients from the whole blood of healthy volunteers. Then, CD4^+^ T cells were purified using a CD4^+^ T Cell Isolation Kit (Miltenyi Biotec, Bergisch-Gladbach, Germany). All the cells mentioned above were cultured in RPMI1640 supplemented with 10% heat-inactivated FBS, 10 mM HEPES, 2 mM L-glutamate and 1% penicillin/streptomycin at 37 °C in humidified atmosphere (5% CO_2_).

Human embryonic kidney 293 (HEK 293) cells were cultured in the same condition as described above. HEK 293 cells were transiently transfected with appropriate cDNA plasmids using Lipofectamine LTX and PLUS (Invitrogen) method as described previously^50^. The cDNA encoding human Kv1.2 (from Prof. Yingliang Wu, Wuhan University, Wuhan, China), human Kv1.3 (generously provided by Dr. Garcia Maria, Merck & Co. Inc., West Point, PA, USA) and human Kv1.5 (from Prof. Guirong Li, Hongkong University, Hongkong, China) were subcloned into the vector of pIRES2-EGFP (Clontech, USA). Point mutations were introduced into the Kv1.3 channel (H399A, V417A) and subcloned into the pRc/CMV plasmid expression vector, which were kindly given by Prof. Yingliang Wu, Wuhan University, Wuhan, China. All plasmids were verified by DNA sequencing before expression. Currents were recorded 48 h after transfection.

### Electric recordings and analysis

The Jurkat T cell-line, human PBTCs and HEK 293 cells were used for the patch-clamp electrophysiological recordings. All studies were performed in the whole-cell recording mode at room temperature as described in our previous studies^19^,^20^,^31^. The external solution contained (in mM): 137 NaCl, 4 KCl, 1 MgCl_2_, 1.8 CaCl_2_, 10 Glucose and 10 HEPES, adjusted to pH 7.4 with NaOH. The pipette solution for Kv1.3 channel currents recording (in mM) was 130 KCl, 1 MgCl_2_, 5 EGTA, 5 Mg-ATP, and 10 HEPES, adjusted to pH 7.2 with KOH. For K_Ca_ recording, the pipette solution contained (in mM) 130 potassium aspartate, 10 EGTA, 8.55 CaCl_2_, 2.08 MgCl_2_, and 10 HEPES, adjusted to pH 7.2 with KOH. In some studies of K_Ca_, the external solution was replaced by the K^+^ Ringer solution, in which all NaCl was replaced by KCl. The depolarization pulse protocol was described in the corresponding results and Figure Legends. The pClamp9.0 and Origin 8.5 software were used for the data acquisition and analysis. The equations used were the same as previously described^19^ to obtain the IC_50_ (drug concentration required for the 50% inhibition of the channel currents), Hill coefficient, inhibition%, half-maximum voltage (*V*_1/2_) and slope factor (*k*) for activation or inactivation, and the reciprocal of the slope (*K*_*i*_).

### Quantitative real-time PCR

Jurkat T cells were seeded and incubated with 10, 30, 100 μM Lovastatin for 24 h, The control group was treated with the corresponding amount of DMSO. Total RNA was isolated from Jurkat T cells using trizol reagent (Invitrogen, CA, USA). cDNA synthesis was performed with RT reagent Kit (Takara Bio., Shiga, Japan). The primers used for the Kv1.3 and GAPDH are as previously described^31^. The real-time PCR Kit (Takara) was used and the reactions were performed in Stepone ABI system. GAPDH was applied as an internal reference. In all experiments, negative controls were included in the absence of the reverse transcriptase. The relative expression quantity 2^−ΔΔCt^ value was calculated to reflect the differences among the groups.

### Western blotting

For the detection of Kv1.3 protein expression, Jurkat cells were treated with 10, 30, 100 μM Lovastatin for 24 h. For the measurement of the NFAT1 and NF-κB activities, Jurkat cells were stimulated with 5 μg/mL PHA and 80 nM PMA for 4 h with or without 10, 30, 100 μM Lovastatin pretreatment for 30 min. Next, the Jurkat cells were collected, and then, the lysate was prepared and analyzed as previously described^19^. The primary antibodies against Kv1.3 (1:500, Alomone), NFAT1 (1:500, Genetex, Irvine, CA, USA), phospho-NF-κB p65/p50 (1:1000, Cell Signaling Technology, MA, USA) and GAPDH (1:2000, Abcam, MA, USA) were applied in our experiments. GAPDH was used as the internal reference. The protein expression levels were measured by enhanced chemiluminescence and analyzed with Image Lab Software.

### Intracellular Ca^2+^ measurement

Jurkat T cells were loaded with 2 μM Fluo-4 AM for 30 min at room temperature, washed twice, and then seeded into the 96-well plate. Lovastatin at 0, 10, 30, 100 μM was applied for 30 min incubation. Thapsgargin (TG, 1 μM) was used to induce Ca^2+^ release in Ca^2+^ free Ringer’s solution, then, 2 mM CaCl_2_ was added for stimulating Ca^2+^ influx. The Ca^2+^ free Ringer’s solution contained (in mM): 155 NaCl, 4.5 KCl, 3 MgCl_2_, 10 Glucose, 5 HEPES, 1 EGTA. With the Enspire Multimode Plate Reader, cells in 96-well plate were illuminated at 488 nm and the fluorescence emissions at 525 nm were captured at 10 s intervals. The index ΔF/F_0_ was calculated to represent the average Ca^2+^ response (ΔF = F−F_0_, F_0_ is the mean value of the background fluorescence).

### Measurement of cell proliferation

Cell proliferation was measured using the Cell Counting Kit-8 (CCK-8) according to the manufacturer’s instructions. Briefly, PBTCs were seeded into 96-well plate in 200 μL medium (5 × 10^5^ per well). Every group contained 4-5 duplicates. PBTCs were pre-incubated with 0, 10, 30, 100 μM Lovastatin for 30 min, then, 1 μg/mL soluble purified anti-CD3 and anti-CD28 antibodies were applied to stimulate T cells for 3 days. 20 μL CCK-8 solution was added to every well for the last 2 h. The optical density (OD) value of every well at 450 nm was measured. The relative cell number was calculated as the percentage of (OD_*test*_−OD_*blank*_)/(OD_*control*_−OD_*blank*_), in which OD_*test*_ is the OD value of wells exposed to Lovastatin or antiCD3/CD28 antibodies, OD_*control*_ is the OD of the control sample and OD_*blank*_ is the OD of wells without PBTCs.

### IL-2 production measurement

Jurkat cells were pre-treated with 0, 10, 30 100 μM Lovastatinin the absence or presence of 1 mM Mevalonate for 30 min. Then the cells were stimulated with 5 μg/mL PHA and 80 nM PMA for 24 h. The supernatants were collected, and the IL-2 production was measured with an ELISA kit following the manufacturer’s instructions. Each experiment was repeated at least 3 times in duplicate.

### Knockdown of Kv1.3 expression with small interfering RNA

The Jurkat T cell-lines stably transfected with lentivirus-delivered negative control (NC)-siRNA or Kv1.3-siRNA were provided by Genepharma Company (Shanghai, China). Western blotting has been performed to confirm the efficiency of siRNA knockdown^31^. The cell-lines were treated as described above and the supernatants were collected for IL-2 production measurement.

### Statistical Analysis

All results are delivered as mean ± SEM. The significance of differences before and after drug application was evaluated using paired t-test. Comparisons between groups were accomplished by analysis of variance with Turkey’s post-test. Significance was set at P < 0.05.

## Additional Information

**How to cite this article**: Zhao, N. *et al*. Lovastatin blocks Kv1.3 channel in human T cells: a new mechanism to explain its immunomodulatory properties. *Sci. Rep*. **5**, 17381; doi: 10.1038/srep17381 (2015).

## Supplementary Material

Supplementary Information
