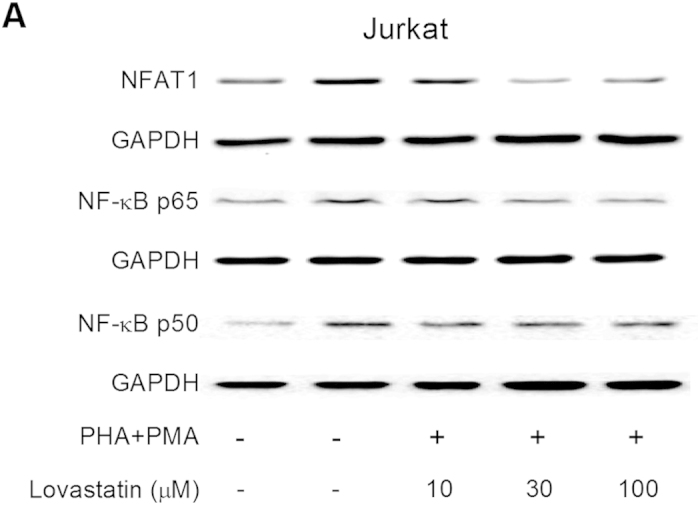# Corrigendum: Lovastatin blocks Kv1.3 channel in human T cells: a new mechanism to explain its immunomodulatory properties

**DOI:** 10.1038/srep21655

**Published:** 2016-03-10

**Authors:** Ning Zhao, Qian Dong, Cheng Qian, Sen Li, Qiong-Feng Wu, Dan Ding, Jing Li, Bin-Bin Wang, Ke-fang Guo, Jiang-jiao Xie, Xiang Cheng, Yu-Hua Liao, Yi-Mei Du

Scientific Reports 5: Article number: 17381; 10.1038/srep17381 published online: 11302015; updated: 03102016

This Article contains errors in Figure 3 and Figure 7a.

In Figure 3A, 3B, 3C, 3D and 3E the current labels were incorrectly provided.

In Figure 7a, the GAPDH Western blot bands were omitted from each corresponding target NFAT1 and NF-κB p65.

The correct Figures 3 and 7a appear below as Figs 1 and 2 respectively.

## Figures and Tables

**Figure 1:**
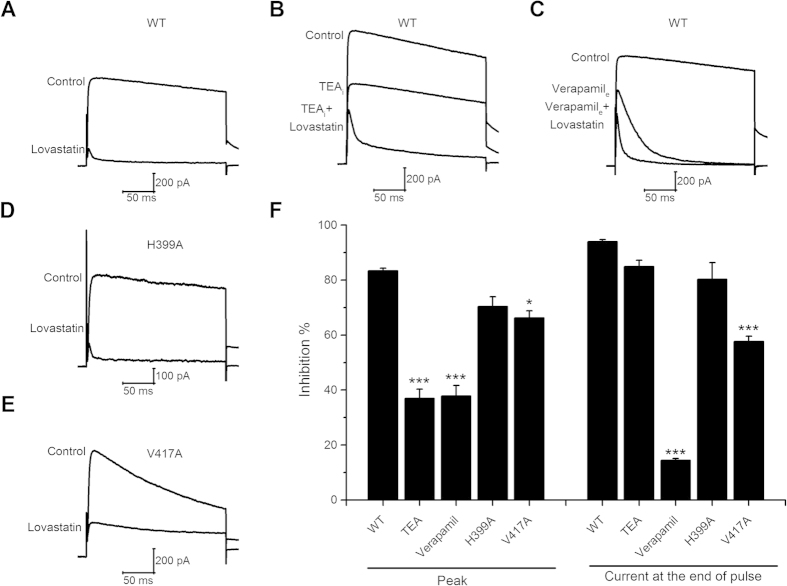


**Figure 2:**