# Supplementary material for: Lovastatin blocks Kv1.3 channel in human T cells: a new mechanism to explain its immunomodulatory properties
Source: Sci Rep. 2015 Nov 30;5:17381. doi: 10.1038/srep17381 (PMC4663632; doi:10.1038/srep17381)
Supplement: Supplementary Information [file srep17381-s1.doc]

**Lovastatin blocks Kv1.3 channel in human T cells: a new mechanism to explain its immunomodulatory properties**

**Ning Zhao^1#^, Qian Dong^1#^, Cheng Qian^1#^, Sen Li^2^, Qiong-Feng Wu^1^, Dan Ding^1^, Jing Li^1^, Bin-Bin Wang^1^, Ke-fang Guo^3^, Jiang-jiao Xie^1^, Xiang Cheng^1^, Yu-Hua Liao^1^, Yi-Mei Du^1^***

^1^ Research Center of Ion Channelopathy, Institute of Cardiology, Union Hospital, Tongji Medical College, Huazhong University of Science and Technology, Wuhan 430022, China.

^2^ Institute of Urology, Union Hospital, Tongji Medical College, Huazhong University of Science and Technology, Wuhan 430022, China.

^3^ Department of anesthesiology, Zhongshan Hospital, Fudan University, Shanghai 200032, China

# These authors contributed equally to this work.

***** Corresponding author: Yi-Mei Du E-mail: [yimeidu@mail.hust.edu.cn](mailto:yimeidu@mail.hust.edu.cn)

**Figure S1 Effects of Lovastatin on human cloned Kv1.x potassium channels transfected in HEK 293 cells.**

Currents were elicited by 200-ms depolarizing voltage steps at +40 mV from a holding potential of -80 mV every 10 sec. **A-C.** Representative current traces in the absence (control) or presence of 30 μM Lovastatin on Kv1.3 (*A*), Kv1.2 (*B*) and Kv1.5 (*C*) channels, respectively. **D.** Average inhibition% of peak and the end of pulse currents by 30 μM Lovastatin.
